# Material-Preserving Extrusion of Polyamide on a Twin-Screw Extruder

**DOI:** 10.3390/polym15041033

**Published:** 2023-02-19

**Authors:** Christoph Schall, Matthias Altepeter, Volker Schöppner, Sven Wanke, Marina Kley

**Affiliations:** 1 Kunststofftechnik Paderborn, Paderborn University, 33098 Paderborn, Germany; 2 Coatings, Materials & Polymers, Paderborn University, 33098 Paderborn, Germany

**Keywords:** extrusion, polyamide, chain extender, molar degradation, polymerization, recycling

## Abstract

In the context of plastics recycling, plastics are processed several times. With each new melting and extrusion the plastic is damaged, which can have a negative effect on product properties. To counteract material damage, special additives such as chain extenders can be used, which are intended to lead to post-polymerization during processing. A linear chain extension is important here, as branching and crosslinking can lead to uncontrolled changes in the plastic’s properties. To investigate the suitability of specialized linear chain extenders for polyamides, a polyamide-6 was processed several times and the molar mass distribution was evaluated after each extrusion cycle. Three series of tests were carried out. First, the plastic was regranulated five times without additives and twice with different concentrations of chain extenders on a twin-screw extruder. The results of the study show that not only can molar mass degradation be prevented with the appropriate additive, it is even possible to achieve a material buildup during processing. In our experiments, the polydispersity of the molar mass distribution remained nearly identical despite multiple extrusions. Thus, reactive extrusion makes it possible for the corresponding plastics to be processed several times without the molar mass decreasing. If a sufficiently pure material flow can be ensured during recycling, the number of possible reprocessings of the plastic can be significantly increased without the need to add virgin material.

## 1. Introduction

A major topic of current interest is the drive towards a circular economy. In order to save new material, or even to completely dispense with it, one focus is the recycling process. Although the term recycling is used frequently, it often refers to downcycling, in which a material is reused in applications with lower mechanical or optical requirements. However, it is important for the future to design and control the recycling processes in such a way that actual recycling can be achieved without loss of quality. This article is not about compound separation or the provision of a pure material flow, as these are mainly construction-related challenges; rather, we examine the processing of plastics in the context of polyamide-6.

The challenge of recycling in terms of processing is the damage incurred by the molar structure of the polymer, as this can negatively affect product properties [1,2,3]. However, as the demands on the properties of plastic products do not decrease, recycled material is often mixed with virgin material to reduce the influence of such damage [1,2,3]. Alternatively, recycled material can be used solely in products with lower mechanical or optical requirements [1,2,3]. However, this does not represent progress towards a circular economy, as the number of reprocessing cycles possible for a plastic is severely limited by its molar mass, meaning that its mechanical and possibly optical properties deteriorate with each new processing operation. If plastics are to be reused as often as possible, they must not degrade on a chemical level in relation to their polymer length if consistent product properties are to be guaranteed.

The change in molar mass during processing is another area of interest for recycling, as the reuse of in-house production scrap leads to multiple processing. This problem applies to all thermoplastics, as even with a pure material stream molar degradation due to multiple extrusion can lead to deterioration of product properties; examples of relevant experimental data can be found in [4,5,6,7,8]. Scientific research on this topic has only been conducted in isolated cases, first beginning in the late 1980s and increasing over the last two decades.

For example, Takeuchi et al. [9] worked first and foremost to specifically influence the recycling process with regard to avoiding damage, while Tzoganakis et al. [10] started by looking at the residence time spectrum as a basis for reactive extrusion with respect to the degradation behavior of polypropylene.

The extent to which the molar structure is damaged depends on the processing conditions [11], among other things. However, it is possible to influence and increase the molar mass through controlled post-polymerisation by means of reactive extrusion with the aid of chain extenders, which ideally should be adapted to the corresponding polymer type [12,13]. There are various chain extenders; these differ at the molar level, and act differently based on their function [13]. In addition to purely building up the molar mass, the type of post-polymerisation is important. Crosslinking and branching in polymer chains leads to a significant increase in molar mass; however, in the case of otherwise linear polymer chains in the virgin material, the change in microstructure can leads to uncontrolled changes in the properties of the plastic, which in turn has a detrimental effect on product properties.

From an economic point of view, recycling only becomes interesting if cost savings can be realized. For this reason, polyamide is examined in this article, as it is significantly more expensive than polyolefins and is used in large quantities due to its advantageous properties. In the group of polyamides, macromolecules are formed through polycondensation; as an equilibrium reaction between water and polyamide, this enables both shortening and extension of the chain. As stated earlier, the polyamide can be post-polymerised in order to prevent the general chain shortening brought on by a further processing step in the recycling cycle. Previous studies have found that the viscosity can be increased 10 to 100 times by reactive extrusion [11].

Preliminary studies by the chair for plastics processing KTP (Kunststofftechnik Paderborn) have dealt with changes in the molar mass of polyolefins during single-screw extrusion [14,15,16] and twin-screw extrusion [17,18] as well as the degradation of PET on a twin-screw extruder [19,20]. The local process events in the extruder were characterised by the local temperature, shear load, and residence time. Ongoing studies are currently dealing with measuring the sensitivity of materials to molar degradation, as very different results can occur even with similar materials.

**Figure 1:**
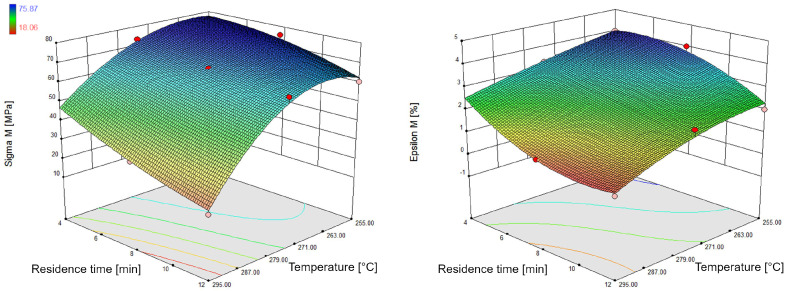
Tensile strength (**left**) and elongation at break (**right**) of a damaged PA6.6 (FRIANYL A63 RV0) specimen at 0.044% moisture content.

Preliminary tests were carried out in which multi-purpose test specimens were moulded from polyamide 6.6 and examined by means of tensile tests in accordance with DIN EN ISO 527. The cylinder temperature of the injection moulding machine and the average residence time of the plastic in the plasticising unit were varied. The maximum tensile stress and the elongation at break of the specimens are shown in Figure 1. Six multi-purpose test specimens per test point were examined.

It can be clearly seen that with longer residence times and higher cylinder temperatures, the mechanical properties are impaired. This can be explained by a change in the chemical structure of the plastic leading to molar degradation. The stronger the molecular degradation, the lower the tensile strength and elongation at break.

The investigations presented in this paper serve to provide a better understanding of the degradation and post-polymerisation behaviour of polyamides for modern linear chain-extenders. Our hope is that in the future, real recycling mass flows with their corresponding impurities can be considered and processed in a molar mass-preserving manner, making multiple processing possible while retaining important product properties. This is critical for the goal of completely closed material loops, which is a major and necessary innovation in the context of the future circular economy, and will be further addressed in upcoming studies.

## 2. Materials and Methods

### 2.1. Used Materials

The plastic investigated was PA-6 Ultramid B3S from BASF, a non-reinforced low-viscosity polyamide. This plastic is mainly used for thin-walled moulded parts. Because the mechanical properties of the plastic are particularly important for these thin-walled parts, any deterioration of its mechanical properties must be avoided [21,22] to ensure consistent component quality even after several recycling cycles. According to ISO 307, the material is characterised by an MVR of 160 cm${}^{3}$/10 min (5 kg/275 °C) and a viscosity number of 145. This results in a resulting zero-shear viscosity of about 220 Pa s at 260 °C [23].

The Bruggolen M1251 chain extender [24] from L. Brüggemann GmbH and Co. KG was used as additive. This is a highly reactive chain extender based on a modified polyamide. The post-polymerisation achieved thereby should be linear, without any branching or cross-linking, to ensure that the molar mass distribution is maintained. The dosage level of the grade is 0.5–2.0%.

### 2.2. Processing

Three test series were carried out to investigate the degradation and post-polymerisation behaviour of the polyamide. In each test series, the polyamide was regranulated five times following the equipment and process parameters described in the following paragraphs: once without chain extender, once with 0.5 mass-%, and once with 1.0 mass-% chain extender. In this way, the influence of the chain extender on the molar mass was observed relative to the same processing with no chain extender. The set mass throughput was 15 kg/h for all test points. The polyamide was pre-dried for 4 h at 80 °C in a dry air dryer before each processing according to the manufacturer’s specifications.

**Figure 2:**
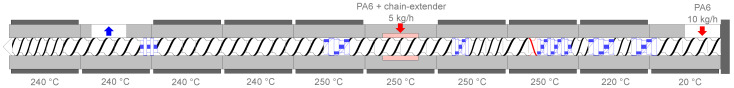
Design of the twin screw extruder.

The tests were carried out on a 25 mm twin-screw extruder (ZSK 25 P8.2E WLE from Coperion Werner & Pfleiderer) with associated dosing, degassing, and control unit. The design of the twin-screw extruder and the temperature settings are shown in Figure 2. The detailed screw design is listed in Table A1. For the processing parameters, we selected were a screw speed of 500 rpm in order to deliberately reflect high shear and the resulting accelerated aging and molar mass degradation caused by multiple processings.

First, 10 kg/h of Ultramid B3S were fed in gravimetrically through the feed in the first cylinder element. The design of the twin screw, especially the kneading blocks, ensures that the 10 kg/h PA6 in the first feeding section is completely melted before the second feeding of the chain extender begins. The remaining 5 kg/h, with the corresponding mass proportion of the chain extender, was then added gravimetrically as a dry blend at the second feed point, which is a side feeder with venting. The later feeding represents a typical compounding process, and additionally ensures a more homogeneous blending of the chain extender into the already existing melt from the first material feeding. The later dosage prevents the reaction from occurring too early. If the chain extender were added in the first stage, this would lead to an increase in molar mass and viscosity; however, the kneading blocks and resulting high shear input would cause a counterproductive increase in molar mass degradation. For this reason, the chain extender was added to the melt and mixed in. In addition, low-molar components (e.g., water vapour) can escape through venting. This is advantageous, as the presence of water has a negative influence on the chemical reaction. In the second-to-last cylinder segment, the melt is additionally freed from low-molar components by means of vacuum degassing.

Re-granulation was carried out using an ECON EUP50 underwater pelletiser with a three-hole plate and a three-knife head. A stable granulation process was achieved by tempering the start-up valve and die plate to 240 °C and the water to 25 °C. The pelletising speed was set to 1200 rpm and the dryer speed to 2500 rpm, which made it possible to produce homogeneous large granules at a constant rate over time.

### 2.3. Measurement

The target parameter of our investigations was the molar mass distribution, as this allows for a full picture of the chemical structure and the corresponding changes due to both molar degradation and post-polymerisation.

Material-preserving extrusion implies the preservation of the chemical structure, and as such, the molar mass distribution. Damage caused by frequent processing and high shear input breaks the chains, causing the molar mass distribution to shift towards lower molar masses; chain extenders are used to counteract this, ensuring post-polymerisation and a shift in the molar mass in the direction of higher values. The additive we used here is a linear extender that results in little to no branching or cross-linking, which, while increasing the molar mass, can negatively influence the resulting material properties.

Gel permeation chromatography (GPC) is a type of size-exclusion chromatography (SEC), and is considered the state-of-the-art for investigating molar mass distribution. This is one of the few methods that can be used to determine the molar mass distribution of polymers. However, as is a relatively expensive and time-consuming measurement method, faster and cheaper methods are often used. These only determine the correlation of the average molar mass, and cannot describe the molar mass distribution. Measurement of the molar mass distribution is, however, important with regard to the investigation of post-polymerization behavior when using chain extenders, as it is only in this way that statements can be made about whether undesirable branching or crosslinking occur.

Each test point was kept stationary when the plastic is regranulated. At each re-granulation, a material sample was taken and the molar mass distribution was determined three times by means of GPC measurements. From this, the change in the molar mass distribution, number average (${\bar{M}}_{n}$) molar mass, and mass average (${\bar{M}}_{w}$) molar mass were determined and compared with a statistical certainty, allowing statements about the degradation and post-polymerisation processes to be made. In addition to the GPC measurements, Differential Scanning Calorimetry (DSC) measurements, a much faster and cheaper measurement method, were performed. In this case, the melting temperature of the samples was investigated, with lower molar mass leading to lower expected melting temperatures. This second method was used as a comparison to investigate the extent to which the DSC measurements correlated with the GPC measurements with regard to statement about changes in molar mass.

## 3. Results

**Figure 3:**
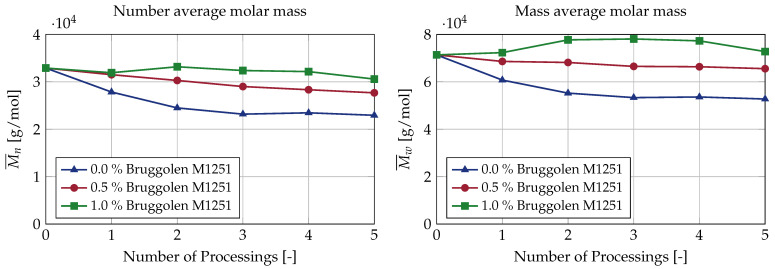
Number and mass-average molar mass over five extrusion cycles for different concentrations of the chain extender.

The number average and mass average molar masses of the plastic samples are shown in Figure 3. The molar mass in g/mol is plotted against the number of processing cycles. The standard deviations of the three GPC measurements carried out in each case are very small, and are plotted in the figures as error bars. Detailed results can be found in tabular form in Table A2.

The molar degradation is very clearly visible in the figure. During re-granulation without addition of the chain extender, a continuous reduction in the number and mass-average molar mass can be seen up to the third processing cycle. After the fourth re-granulation, both the number average and the mass average of the molar mass increase slightly. After the fifth processing, however, the molar mass drops again to a level below that of the third re-granulation. After the third re-granulation, there is only a very slight change in the molar mass distribution, and the degradation of the material almost stagnates. This can be explained by the fact that as the polymer chains become shorter, the mechanical stress on the polymer chains decreases due to the shear and temperature during processing in the twin-screw extruder. These results contrast with the test series incorporating the chain extender. With the addition of 0.5% chain extender, a continuous decrease can be seen in the number average molar mass, while the molar mass remains significantly higher than in the reference process without any chain extender. For the mass average molar mass, the course is almost constant over the number of processing cycles, and only decreases slightly due to the multiple re-granulations. The third test series, with addition of 1.0% chain extender, shows an even more different picture. The number average molar mass remains almost constant over all five regranulation processes. In contrast, there is a slight increase in the mass average molar mass. For the third processing step, the mass average molar mass is 9.4% higher than the molar mass of the unprocessed plastic. After the fifth processing step, the mass average molar mass is still 2.0% higher.

In summary, both the number average and mass average molar mass decrease during processing without any chain extender. With a low addition of 0.5% chain extender, the number average molar mass decreases, while the mass average molar mass remains approximately constant. With a standard dosage of 1.0% chain extender, the number average molar mass remains approximately constant, while the mass average molar mass increases.

**Figure 4:**
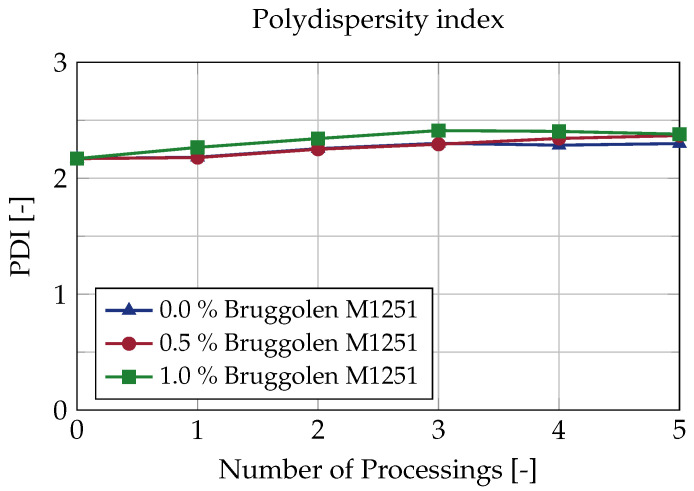
Polydispersity index over five extrusion cycles for different concentrations of chain extender.

In order to better illustrate this fact, the polydispersity index of the molar mass distribution is shown in Figure 4. The polydispersity index represents a measure of the width of the molar mass distribution, with lower the polydispersity indicating a more uniform length of the individual polymer chains. A molar mass distribution already arises during the polymerisation of the plastics, as slight statistical inequalities in the chemical process result in different chain lengths. A narrow distribution results in a narrower thermal softening range, while a wide distribution results in a higher proportion of low-molecular polymer chains, which act as a “lubricant” during processing. Furthermore, the tendency towards crystallisation increases with lower chain lengths, whereas high molecular chains result in improved mechanical properties, among other things. Because both short and long polymer chains influence the material properties, the polydispersity should remain unchanged in the case of constant product properties [25].

The polydispersity index is calculated from the ratio of the mass average molar mass to the number average molar mass:

$$\mathrm{Polydispersity}\;\mathrm{index}=\frac{{\bar{M}}_{w}}{{\bar{M}}_{n}}$$

Based on these results, no significant changes can be detected in the polydispersity index. For all three test series, there is only a slight trend towards a higher index. While the virgin material has a polydispersity index of 2.17, this value increases to 2.23 after five-fold re-granulation without the use of chain extenders and to 2.38 with the addition of 1.0% chain extender. Thus, post-polymerisation has only a very small influence on the polydispersity index.

**Figure 5:**
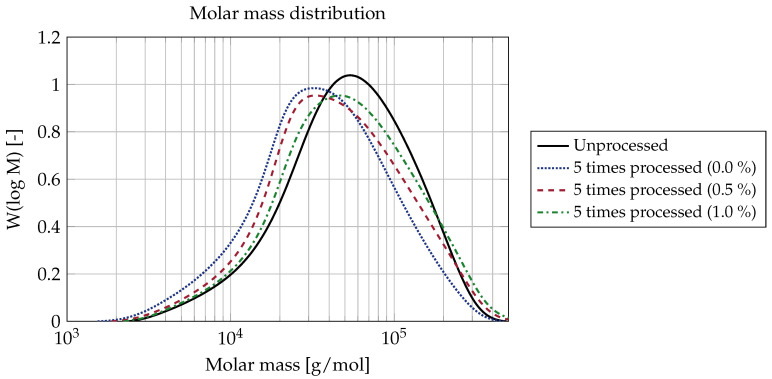
Molar mass Distribution for the virgin plastic and the samples subjected to five-fold processing.

In order to show this more clearly, the molar mass distributions of the virgin plastic and the plastic after five-fold processing in the three test series are shown in Figure 5. The unprocessed plastic shows the narrowest molar mass distribution. The use of the chain extender does not seem to have any influence on the shape of the distribution. A monomodal distribution can be seen in all measurements, and that no branching or cross-linking can be observed. Without the addition of chain extender, the proportion is higher at low molar masses compared to the other measurement curves, and is lower at high molar masses. With increasing dosage of the chain extender, the proportion of low molar masses decreases and the proportion of high molar masses increases. The peak of the most frequently occurring molar mass moves towards higher molar masses with increasing dosage of the chain extender.

Furthermore, DSC measurements were carried out for the virgin material as well as for the five-fold processed samples from the three test series. However, no clear results could be derived, as the changes in the melting temperature are very small and show no clear trend. Although the measurement of material degradation by DSC measurements is significantly faster and cheaper than GPC measurements, it is not a suitable method for assessing material degradation or even post-polymerization. The measured melting temperatures are shown in Table 1.

## 4. Discussion

The results of this study show that using chain extenders in a closed polyamide material cycle can retain the mean molar mass. Without the addition of chain extenders, material degradation appears to stagnate at a certain level; however, it can be assumed that the material properties are already significantly degraded at this point, precluding use the degraded material for recycling. For molar mass-preserving processing, the use of chain extenders is absolutely necessary in order to maintain a molar mass distribution that is as constant as possible by means of post-polymerization. By dosing with a chain extender, the molar mass can be adjusted without any significant change in molar mass distribution. The polydispersity index only increases slightly, which is the case even without the use of chain extenders. The main advantage of the method presented in this paper is the determination of the total molar mass distribution on the basis of GPC measurements. In this way, the relative proportions of high and low molecular weight polymer chains can be seen, as well as whether the distribution is strongly altered by the addition of chain extenders, such as in the cases of undesirable crosslinking or branching. Thus, for the investigated chain extender it is apparent that the molar mass distribution remained monomodal and the polydispersity was hardly influenced by the chain extender, indicating the absence of any undesired crosslinking or branching.

For future studies, it would makes sense to take a broader approach that incorporates the resulting product properties, as only molar mass was investigated in this study. For a practical evaluation, it is necessary to investigate material properties such as the mechanical properties of multiple re-granulated polyamides. In addition to the mechanical properties, a higher proportion of high molecular weight chains can have an influence on the rheological properties, and thereby on processing, as the flow behavior can be significantly influenced even with similar mean molar masses. Nonetheless, the results we have presented suggest that the addition of chain extenders and the resulting higher molar mass can allow for a higher number of recycling cycles while at the same time ensuring better product quality.

## Figures and Tables

**Table 1:** Measured melting temperatures.

Test Point	Melting temperature
virign plastic	222.87 °C
5× processed (0.0%)	222.14 °C
5× processed (0.5%)	223.62 °C
5× processed (1.0%)	221.71 °C

## Data Availability

Not applicable.

## Appendix A

**Table A1:** Listing of the screw elements of the twin screw extruder.

Pos.	mm	Name
1	18	36/18
2	54	36/36
3	90	36/36
4	126	KB45/5/36
5	150	24/24
6	186	KB45/5/36
7	210	24/24
8	234	KB45/5/24
9	258	KB45/5/24
10	270	24/12 left
11	306	36/36
12	330	24/24
13	354	24/24
14	378	KB45/5/24
15	414	36/36
16	450	36/36
17	486	36/36
18	522	36/36
19	558	KB45/5/36
20	576	36/18
21	612	36/36
22	630	36/18
23	666	36/36
24	702	36/36
25	738	36/36
26	756	36/18
27	792	36/36
28	816	KB90/5/24
29	852	36/36
30	888	36/36
31	924	36/36
32	948	24/24
33	972	24/24
34	996	24/24

## Appendix B

**Table A2:** List of the molar masses of all measurements (values in g/mol).

Test Point	${\bar{M}}_{n,1}$	${\bar{M}}_{w,1}$	${\bar{M}}_{n,2}$	${\bar{M}}_{w,2}$	${\bar{M}}_{n,3}$	${\bar{M}}_{w,3}$	${\bar{M}}_{n,\mathrm{mean}}$	${\bar{M}}_{w,\mathrm{mean}}$
virign plastic	33,307	72,005	33,066	71,528	32,318	70,529	32,897	71,354
1× processed (0.0%)	28,103	61,465	27,673	60,380	27,750	60,343	27,842	60,729
2× processed (0.0%)	23,388	53,512	25,209	56,758	24,889	55,409	24,495	55,226
3× processed (0.0%)	22,175	51,363	23,692	54,249	23,673	54,382	23,180	53,331
4× processed (0.0%)	23,506	53,336	23,395	53,813	23,472	53,651	23,458	53,600
5× processed (0.0%)	22,822	52,069	22,893	52,560	23,103	53,610	22,939	52,746
1× processed (0.5%)	30,665	66,300	31,669	69,119	32,157	70,356	31,497	68,592
2× processed (0.5%)	29,477	67,132	30,441	68,253	30,938	69,080	30,285	68,155
3× processed (0.5%)	28,258	65,001	28,938	66,837	29,803	67,674	29,000	66,504
4× processed (0.5%)	27,707	65,745	28,174	65,360	29,093	67,954	28,325	66,353
5× processed (0.5%)	27,497	64,486	27,695	65,600	27,833	66,537	27,675	65,541
1× processed (1.0%)	31,740	71,777	32,306	73,160	31,694	71,972	31,913	72,303
2× processed (1.0%)	33,250	77,131	32,727	78,287	33,530	77,644	33,169	77,687
3× processed (1.0%)	32,322	79,016	31,864	76,030	32,945	79,171	32,377	78,072
4× processed (1.0%)	33,187	79,174	31,664	76,144	31,595	76,498	32,149	77,272
5× processed (1.0%)	31,306	73,986	30,716	72,057	29,707	72,229	30,576	72,757
